# Heterojunction α-Co(OH)_2_/α-Ni(OH)_2_ nanorods arrays on Ni foam with high utilization rate and excellent structure stability for high-performance supercapacitor

**DOI:** 10.1038/s41598-019-49138-5

**Published:** 2019-09-04

**Authors:** Shaojie Zhou, Wutao Wei, Yingying Zhang, Shizhong Cui, Weihua Chen, Liwei Mi

**Affiliations:** 1 Center for Advanced Materials Research, Zhongyuan University of Technology, Zhengzhou, Henan 450007 P.R. China; 2 College of Chemistry and Molecular Engineering, Zhengzhou University, Zhengzhou, Henan 450001 P.R. China

**Keywords:** Two-dimensional materials, Nanowires

## Abstract

The practical implementation of supercapacitors is hindered by low utilization and poor structural stability of electrode materials. Herein, to surmount these critical challenges, a three-dimensional hierarchical α-Co(OH)_2_/α-Ni(OH)_2_ heterojunction nanorods are built *in situ* on Ni foam through a mild two-step growth reaction. The unique lamellar crystal structure and abundant intercalated anions of α-M(OH)_2_ (M = Co or Ni) and the ideal electronic conductivity of α-Co(OH)_2_ construct numerous cross-linked ion and electron transport paths in heterojunction nanorods. The deformation stresses exerted by α-Co(OH)_2_ and α-Ni(OH)_2_ on each other guarantee the excellent structural stability of this heterojunction nanorods. Using nickel foam with a three-dimensional network conductive framework as the template ensures the rapidly transfer of electrons between this heterojunction nanorods and current collector. Three-dimensional hierarchical structure of α-Co(OH)_2_/α-Ni(OH)_2_ heterojunction nanorods provides a large liquid interface area. These result together in the high utilization rate and excellent structure stability of the α-Co(OH)_2_/α-Ni(OH)_2_ heterojunction nanorods. And the capacitance retention rate is up to 93.4% at 1 A g^−1^ from three-electrode system to two-electrode system. The α-Co(OH)_2_/α-Ni(OH)_2_//AC device also present a long cycle life (the capacitance retention rate is 123.6% at 5 A g^−1^ for 10000 cycles), a high specific capacitance (207.2 F g^−1^ at 1 A g^−1^), and high energy density and power density (72.6 Wh kg^−1^ at 196.4 W kg^−1^ and 40.9 Wh kg^−1^ at 3491.8 W kg^−1^), exhibiting a fascinating potential for supercapacitor in large-scale applications.

## Introduction

The rapid consumption of non-renewable energy resources and the booming development of mobile electronics and hybrid electric vehicle have aroused intensive attention on green and safe energy storage devices with high specific capacitance and excellent working life^1–5^. Supercapacitors as emerging energy storage devices combine the advantages of conventional capacitors and batteries and possess the high-power density, long cycle life, fast charge/discharge performance, safe reliability and environmentally friendly at the same time, resulting in the explosive-growth attention of researchers^6–10^. Electrode material is recognized as the core component of supercapacitors, which determines the comprehensive performance of supercapacitors^4,11–13^. Therefore, novel electrode materials for supercapacitors emerge in an endless stream in recent years. However, most of the reported electrode materials present poor ionic conductivity, weak electronic conductivity or unstable crystal structure. The first two factors lead to the reversible redox reaction occurring only on the surface of electrode materials, further determining the low utilization rate of the electrode material. The third factor results in a short cycle life^14,15^. These have forced the researchers to improve the utilization rate and structure stability of electrode materials, which is also an enormous challenge.

In recent years, the heterojunction composites have been widely studied in photocatalysis and solar cells^16–18^. It is owing to the construction of heterojunction realizing the advantages complementary and synergistic effects of two different materials^14,19,20^. Inspired by this innovation, the researchers also try to improve the comprehensive performance of electrode materials by building rich heterojunctions inside electrode materials and successfully prepared a series of heterojunction composites for supercapacitors (such as Ni_3_S_2_/Ni(OH)_2_^21–23^, NiS/Ni(OH)_2_^24,25^, NiCo_2_S_4_/Ni(OH)_2_^26–28^ and CoS/Ni(OH)_2_^29^), which combine the high ionic conductivity of Ni(OH)_2_ electrode attributing to its two-dimensional layered crystal structure and the excellent electronic conductivity of nickel sulfide owing to its compact crystal structure and high metallicity of sulfur atoms. These reports provide effective strategies for building novel electrode materials for supercapacitors. However, there arises another puzzle that the specific capacitance of electrode materials in two-electrode system is much lower than that in three-electrode system. This may be due to the fact that high ion and electron conductivity can only be achieved at heterojunctions and β-Ni(OH)_2_ without abundant intercalated anions as steady state Ni(OH)_2_ exhibits no satisfactory ionic conductivity. Therefore, the preparation of electrode materials with both high utilization rate and excellent stability still needs to be further studied, which is also of great significance. Based on previous research, α-Ni(OH)_2_ with abundant intercalated anions between two-dimensional layered crystal structure exhibits much better ionic conductivity than β-Ni(OH)_2_^30^. α-Co(OH)_2_ with the similar crystal structure of α-Ni(OH)_2_ also exhibits excellent ionic conductivity. Besides, α-Co(OH)_2_ possesses ideal electronic conductivity^31,32^. But the anions between layered crystal structures can easily escape, especially in alkaline aqueous environment, which causes the unstable crystal structure of α-Ni(OH)_2_ and α-Co(OH)_2_ and the reduction in the interlamellar spacing^30,33,34^. Meanwhile, the deformation stress is produced by the change of interlamellar spacing. The construction of α-Co(OH)_2_/α-Ni(OH)_2_ heterojunctions can realize the complementarity of their advantages and further improve the utilization of electrode materials. In addition, their deformation stress may achieve synergistic effect and increase the structural stability of each other. So designing a mild strategy to construct the α-Co(OH)_2_/α-Ni(OH)_2_ heterojunction composite is expected to realize an electrode material with high utilization rate and stable structure.

In addition to the ionic and electronic conductivity and structural stability of the electrode material itself, the electronic transmission rate between electrode material and current collector and the ionic diffusion rate between electrode material and electrolyte also have a great influence on the performance of supercapacitor. The former can be achieved by combining electrode materials with highly conductive materials (such as active carbon, carbon nanotube, graphene, metal foam)^6,13,35,36^, especially nickel foam with three-dimensional (3D) conductive framework, light weight, strong mechanical properties, flexibility, which is often used as a template to prepare an integrated electrode material and as the conductive bridge between current collector and electrode material to improve electronic transmission performance^5^. The latter is usually achieved by using micro-nano fabrication techniques to obtain electrode materials with 3D hierarchical structure, which can improve the liquid-connection area between electrode material and electrolyte and further reduce concentration polarization^37^. Therefore, it is of great significance and much anticipated research to fabricate α-Co(OH)_2_/α-Ni(OH)_2_ heterojunction composite with 3D hierarchical structure and assemble it on the surface of nickel foam to obtain integrated electrode materials without binder.

In this work, the α-Co(OH)_2_ nanowires and α-Ni(OH)_2_ nanosheets were assembled successively *in situ* on the surface of nickel foam by the two-step low temperature oscillation method, resulting in the successful preparation of 3D hierarchical α-Co(OH)_2_/α-Ni(OH)_2_ heterojunction nanorods. The complementary advantages of conduction features and synergistic effect of deformation stress between α-Co(OH)_2_ and α-Ni(OH)_2_ endow the as-constructed heterojunction nanorods with high utilization rate and structural stability. The 3D highly conductive performance of nickel foam and 3D hierarchical structure of heterojunction composites construct an efficient electronic and ion transport network around the electrode materials. This ingenious design ensures α-Co(OH)_2_/α-Ni(OH)_2_ heterojunction nanorods with a high capacitance retention rate of single electrode between three-electrode system and two-electrode system (the capacitance retention rate is 93.4% at 1 A g^−1^) and long cycle life (the capacitance retention rate is 123.6% at 5 A g^−1^ for 10000 cycles). In addition, the α-Co(OH)_2_/α-Ni(OH)_2_//AC device also exhibits excellent rate performance (the capacitance retention rate is 70.2% from 1 to 20 A g^−1^) and idea energy density and power density (72.6 Wh kg^−1^ at 196.4 W kg^−1^ and 40.9 Wh kg^−1^ at 3491.8 W kg^−1^). The synthesis procedure is relatively very simple yet allows the formation of heterojunction nanorods with excellent comprehensive performance, which is interesting for large-scale production and utilization in supercapacitors.

## Experimental Section

### Synthesis of the α-Co(OH)_2_ nanowires

0.0436 g of cobalt nitrate and 1 g of urea were put into a mixture of ethanol and distilled water with a ratio of 2:3 and the solution was stirred to mix well. Then, the solution was moved into a test tube of 20 mL and nickel foam was placed in the solution under water bath equipment with vibration at 80 °C for 90 min. After that, surface of the reacted Ni foam was washed many times using distilled water and ethanol, respectively, and dried overnight in a 60 °C oven. The weight of Co(OH)_2_ nanowire was about 1 mg by comparing the weight before Ni foam.

### Preparation of α-Ni(OH)_2_/α-Co(OH)_2_ heterojunction nanorods

The amount of nickel nitrate and urea used in the synthesis of heterojunction nanorods is the same as that of cobalt nitrate and urea, and the reaction temperature and solvent conditions are also similar to that used in the synthesis of cobalt hydroxide. The difference is that the second reaction takes less time than the first, which consume only 30 min. After the same operation and drying as the previous experiment, the weight of α-Co(OH)_2_/α-Ni(OH)_2_ nanorods was about 1.4 mg by subtracting the weight of Ni foam.

### Preparation of activated carbon (AC) electrode and device

Firstly, AC and polyvinylidene fluoride according to the mass ratio of 9:1 were put into a solution of ethanol and isopropyl alcohol with volume ratio of 1:1. Then, the blended liquid was ultrasonic for 30 min in the ultrasonic device so that the solution is distributed evenly. Next, the solution is trickled onto surface of 3D Ni foam by dropwise addition. After dropping the slurry, the Ni foam was dried by blowing in the oven of 60 °C. Next, by subtracting the weight before and after the Ni foam, the mass of the supported activated carbon is about 30 mg. Last, a self-assembling routine is used to design a device from AC and the active materials to test its application.

### Material characterizations

Morphology of the sample were taken using Zeiss Merlin Compact scanning electron microscope (SEM) with own an energy dispersive X-ray spectroscopy (EDS). TEM were verified by a JEOL JEM-2010 transmission electron microscope. X-ray diffraction (XRD) measurements were carried out using a Bruker D8 Advance X-ray powder diffractometer with Cu-Kα irradiation (10°–90°, a scan rate of 0.1° s^−1^ (2θ)). FTIR spectrums of the sample were analyzed using Fourier transform infrared spectrometer equipment (NicoletiS50, Thermo fisher scientific). The chemical feature on the surface of heterojunction was identified by XPS (Producer: Thermo Fisher, model number: K-Alpha).

### Electrochemical characterization

The active material, Hg/HgO electrode and platinum electrode act as working electrode, reference electrode and counter electrode for three-electrode system respectively. AC, membrane and the active materials are blended into a sandwich-like device to evaluate its performance. The Cyclic voltammetry (CV) and electrochemical impedance spectroscopy (EIS) analyses of the all sample were tested using the 660E electrochemical workstation produced by shanghai Chenhua, china. Galvanostatic charge/discharge measurements were assessed by using the CT2001A LAND battery test equipment. The above all tests are performed in 2 M KOH solution.

## Results and Discussion

**Figure 1:**
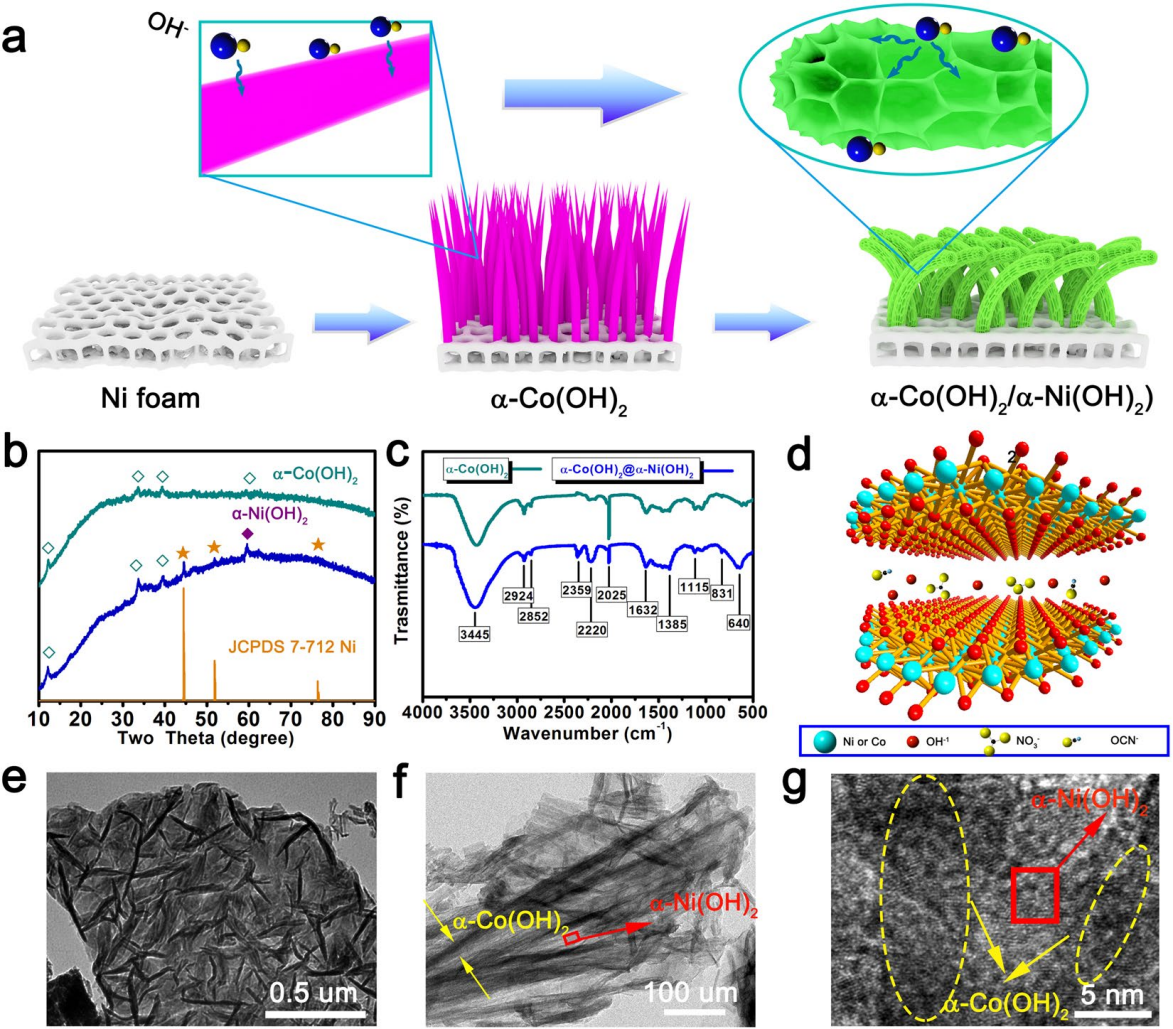
(**a**) Abridged general view of preparation of α-Co(OH)_2_ nanowire and heterojunction α-Co(OH)_2_/α-Ni(OH)_2_, (**b**) corresponding XRD, (**c**) their FTIR spectrum, (**d**) lattice diagram of the intercalated structure, (**e**–**g**) TEM images of heterojunction α-Co(OH)_2_/α-Ni(OH)_2_ nanorods.

Figure 1a offers schematic illustration of α-Co(OH)_2_ nanowires and α-Co(OH)_2_/α-Ni(OH)_2_ heterojunction nanorods preparation. The α-Co(OH)_2_ nanowires as precursors were first fabricated via a mild (water and ethanol) and low-temperature (80 °C) water bath shaking method. Then the α-Ni(OH)_2_ nanosheet is based on the α-Co(OH)_2_ nanowire, the whole covers its surface and further forms α-Co(OH)_2_/α-Ni(OH)_2_ heterojunction nanorods. Compared with smooth nanowires, heterojunction structure with the pleated sheet surface expands the contact between the material and electrolyte, further promoting the ion transport. The diffraction patterns of X-ray diffraction (XRD) are displayed in Fig. 1b. For nanowires sample, the critical position of the peak at about 12.3°, 33.58° and 39.5° are match with (003), (012) and (015) facets of α-Co(OH)_2_ respectively^38^. After the nickel hydroxide coating process, a strengthened peak appeared at 59.4°, which corresponds to (300) crystal plane of α-Ni(OH)_2_^39^. This result confirms the α-Ni(OH)_2_ is successfully bonded to the surface of α-Co(OH)_2_. To fully prove the coexistence of α-Co(OH)_2_ and α-Ni(OH)_2_, the complex was further investigate by XPS test (the specific details are shown in Fig. S1)^40,41^. In addition, the presence of peaks at 44.6°, 51.9° and 76.4° are caused by introducing a small amount of nickel foam due to ultrasonic treatment for samples. The XRD data indicates that the as- fabricated Co(OH)_2_ and Ni(OH)_2_ all demonstrate alike structure with α phase. To further verify the internal structure of the synthesized materials, the FTIR spectrum of pure α-Co(OH)_2_ and α-Co(OH)_2_/α-Ni(OH)_2_ heterojunction hybrid are present in Fig. 1c. The bands located at 2924 cm^−1^ and 2852 cm^−1^, which belongs to the asymmetric and symmetric CH_2_ stretching of alkyl amine^42^. The bands at 3445 cm^−1^ and 640 cm^−1^ are due to the O-H stretching modes of interlayer water molecules and H-bound of OH groups, and the band at 1628 cm^−1^ is proved to the bending vibration mode of the water molecules^43,44^. The band appears at 1382 cm^−1^ is attributed to the existence of interlayer nitrate anion^43^. The 2220 cm^−1^ corresponds to the vibration of interlayer OCN^−1^ anions, which further confirm the presence of α-Ni(OH)_2_ because it only occurs in the example of nickel hydroxide^45^. The strong peaks at 2025 cm^−1^ corresponds to the absorption peak of carbonyl group^46^, and relative intensity of the peak in the heterojunction structure is much weaker, which illustrates that amount of NH_2_COO^−^ anions have been reduced after second reaction. A small loss of anions may help the heterogeneous material with a two-dimensional layered crystal structure opens up more space to facilitate ion of dissociation, leading to high ion transport rate. As can be seen from the schematic diagram of crystal structure of materials (Fig. 1d), the large space between layers obviously facilitates the shuttling and movement of ions, which fully confirms the advantages of layered materials. The TEM images verified α-Co(OH)_2_ nanowires existed within the flake structure of α-Ni(OH)_2_ (Fig. 1e–g), indicating the successful construction of α-Co(OH)_2_/α-Ni(OH)_2_ heterojunction.

**Figure 2:**
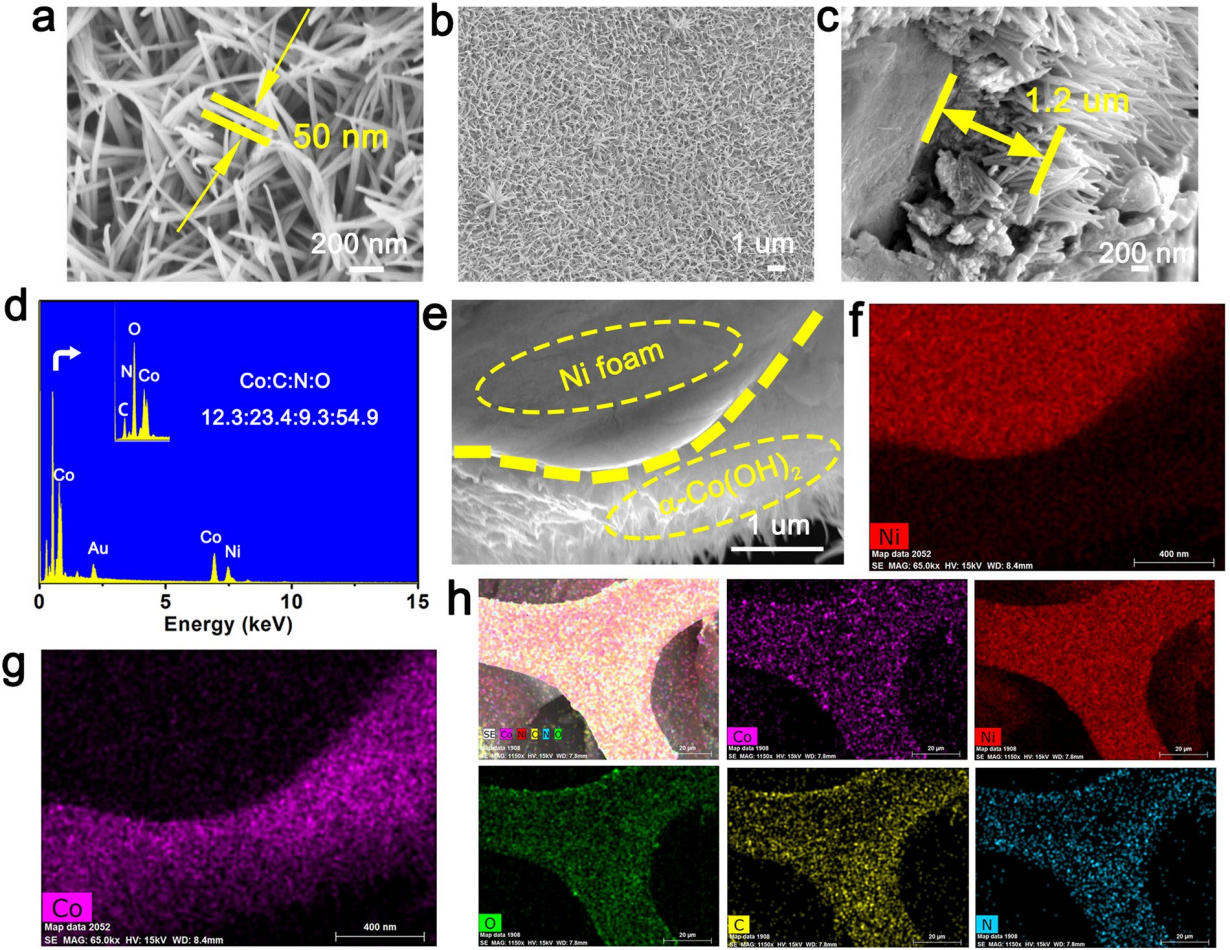
Morphology and elemental characterization of α-Co(OH)_2_ nanowires. (**a**–**c**,**e**) SEM, (**d**) EDS, (**f**–**h**) Mapping.

From the SEM images of α-Co(OH)_2_ (Fig. 2a), we can distinctly observe the ultrafine nanowires (about 50 nm in diameter). Figure 2b shows the low-magnification SEM image, which indicates that the α-Co(OH)_2_ nanowires are attached to the surface of Ni foam. As exhibited in Fig. 1c, the length of nanowires on nickel foam was approximately 1.2 um. Figure 2d shows that energy dispersive spectrum (EDS) image of α-Co(OH)_2_ nanowires. The appearance of characteristic peaks of elements confirms the existence of corresponding elements (Ni, Au, Co, N, C and O). In addition to Au and Ni elements (they come from the gold target and nickel foam, respectively), C and N elements arrives from the anions of the intercalated structure of α-Co(OH)_2_ material, which is consistent with the FTIR spectrum analysis. The Co:C:N:O elements ratio of the as-fabricated α-Co(OH)_2_ nanowires is 12.3: 23.4:9.3:54.9. The molar ratio of C element is higher than that of Co, which indicates that there are abundant anions in the two-dimensional spacing of α-Co(OH)_2_. In addition, the sample α-Co(OH)_2_ and Ni foam can be clearly recognized by SEM and mapping distribution in Fig. 2(e–g), which further indicates that the synthesized cobalt material grows uniformly on the nickel foam. The mapping measurements (Fig. 2h) show that the distribution of the Co, C, N and O elements. The skeleton structure formed by the distribution of the uniform color representing different elements are similar, which illustrates the α-Co(OH)_2_ present uniform distribution on the surface of nickel foam.

**Figure 3:**
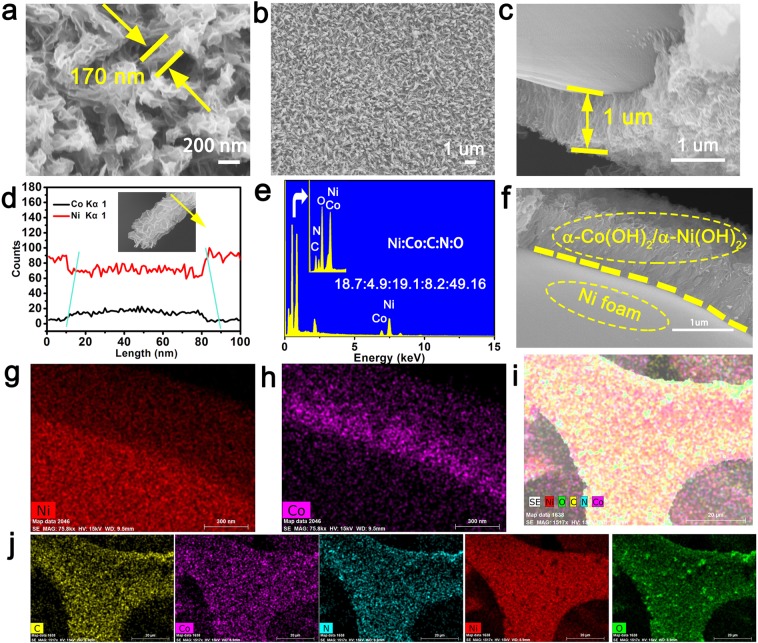
The SEM images, EDX and Mapping of α-Co(OH)_2_/α-Ni(OH)_2_ heterojunction nanorods, (**a**–**c**,**e**) SEM, (**d**) EDX line scanning, (**e**) EDX, (**g**–**j**) Mapping.

After the second reaction, the morphology of the nanowire turned into nanorods which was covered by nickel hydroxide nanoflakes (Fig. 3a). The diameter of nanorods is about 170 nm, which shows significant overall increases in diameter than the nanowires. In addition, surface of the nanorods possesses a plicate morphology, leading to rich ions contact between the surface and electrolyte. To further observe growth and distribution of the material, lower SEM image is displayed in Fig. 3b. Clearly, the α-Co(OH)_2_/α-Ni(OH)_2_ heterojunction nanorods are uniformly supported on the surface of Ni foam. This α-Co(OH)_2_/α-Ni(OH)_2_ heterojunction nanorods material indicated a thickness of approximately 1 um (Fig. 3c), and the length is analogical to that of α-Co(OH)_2_ nanowires. EDX line scanning based nanorods was also detected. The distribution of higher Co and lower nickel element in the center section and lower Co and higher Ni in the edge area further confirms the heterojunction formed by cladding (Fig. 3d). The material that grows on the edge of Ni foam are further analyzed by EDX in Fig. 2e and quantity of the Ni, Co, C, N and O elemental are 18.7:4.9:19.1:8.2:49.2. The constituent of C and N are relatively high and the cause is the same as α-Co(OH)_2_. From Fig. 3g,h we can obtain the element include Co and Ni display similar layout, thus proving uniform arrangement of Ni and Co elements. Furthermore, by analyzing the element scanning results of the nickel-foam skeleton with α-Co(OH)_2_/α-Ni(OH)_2_ heterojunction, which further confirms homogeneity of the growth of heterojunction materials (Fig. 3i,j).

**Figure 4:**
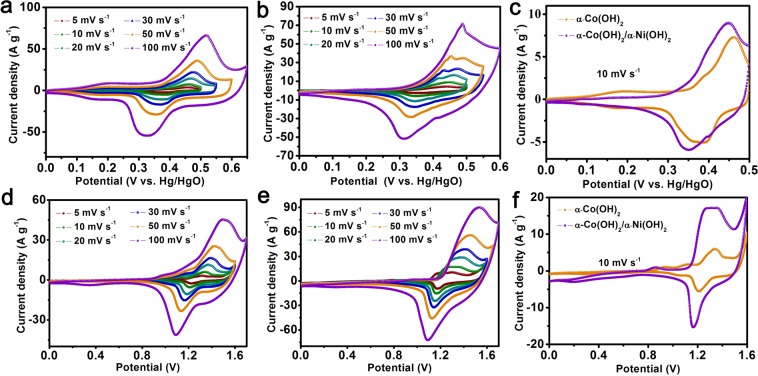
(**a**,**b**) The CV curves of Co(OH)_2_ nanowires and heterojunction α-Co(OH)_2_/α-Ni(OH)_2_ nanorods at various scanning speed in three-electrode system, respectively, (**c**) the corresponding CV curves at 10 mV s^−1^ in three-electrode system, (**d**,**e**) the curves with various scanning speed of Co(OH)_2_ nanowires and α-Co(OH)_2_/α-Ni(OH)_2_ heterojunction in two-electrode system, respectively, (**f**) the CV curves at 10 mV s^−1^ in two-electrode system.

All electrochemical behaviors of the sample as electrode materials are assessed in 2.0 M KOH aqueous electrolyte. The CV curves of α-Co(OH)_2_ (Fig. 4a,d) and α-Co(OH)_2_/α-Ni(OH)_2_ (Fig. 4b,e) heterojunction with different scanning velocities (5 to 100 mVs^−1^) were performed to study the corresponding electrochemical behavior on the surface of the electrode material in three-electrode system and two-electrode system, respectively. All CV curves reveal well peaks shapes, which indicate a behavior with the reversible pseudo-capacitance on the surface of the electrode material (The detailed analysis can be seen in Fig. S2). The process can be proposed as follows^15,30^:

1 $${\rm{Co}}{({\rm{OH}})}_{2}+{{\rm{OH}}}^{-}\leftrightarrow {\rm{CoOOH}}+{{\rm{H}}}_{2}{\rm{O}}+{{\rm{e}}}^{-}$$

2 $${\rm{CoOOH}}+{{\rm{OH}}}^{-}\leftrightarrow {{\rm{CoO}}}_{{\rm{2}}}+{{\rm{H}}}_{2}{\rm{O}}+{{\rm{e}}}^{-}$$

3 $${\mathrm{Ni}(\mathrm{OH})}_{{\rm{2}}}+{{\rm{OH}}}^{-}\leftrightarrow {\rm{NiOOH}}+{{\rm{H}}}_{2}{\rm{O}}+{{\rm{e}}}^{-}$$

When increasing the scanning speed, an obvious phenomenon is that the position of anode peak and cathode peak move a small deviation towards the direction of positive pole and negative pole, respectively, which indicates only a small polarization of surface of the electrode materials occurs during the reversible reaction. Moreover, comparing the peak shapes of their CV curves, The α-Co(OH)_2_/α-Ni(OH)_2_ heterojunction materials provided greater peak area, indicating that heterojunction materials contained higher specific value than α-Co(OH)_2_.

On the other hand, the two oxidation peaks of the α-Co(OH)_2_/α-Ni(OH)_2_ heterojunction finally converge into one peak (Fig. 4b,e) under three-electrode system and two-electrode system, this may due to the enhanced synergy between α-Co(OH)_2_ and α-Ni(OH)_2_ materials. To contrast the change further, the CV curves at sweep rate with 10 mV s^−1^ of α-Co(OH)_2_ and α-Co(OH)_2_/α-Ni(OH)_2_ heterojunction electrode materials in three-electrode system and two-electrode system are exhibited in Fig. 4c,f, respectively. Obviously, the coordinates of the oxidation peak and the reduction peak in their CV are shifted to the left, which can be considered as the result of the coordination between the two materials. Different from α-Co(OH)_2_/α-Ni(OH)_2_ heterojunction, there are two pairs of clear redox peaks for α-Co(OH)_2_ in three-electrode system (Fig. 4c). The first pair peaks are present at 0.21 V and the second pair peaks are located at 0.47 V and 0.38 V, which are corresponding to the processes of the reaction Eqs (1 and 2), respectively. However, α-Co(OH)_2_/α-Ni(OH)_2_ heterojunction electrode materials only display a pair peaks, which due to internal α-Co(OH)_2_ material is not activated and the corresponding current is low. When the scanning speed is increased, the two oxidation peaks can be observed (Fig. 4c), which may be attributed to excitation of α-Co(OH)_2_.

**Figure 5:**
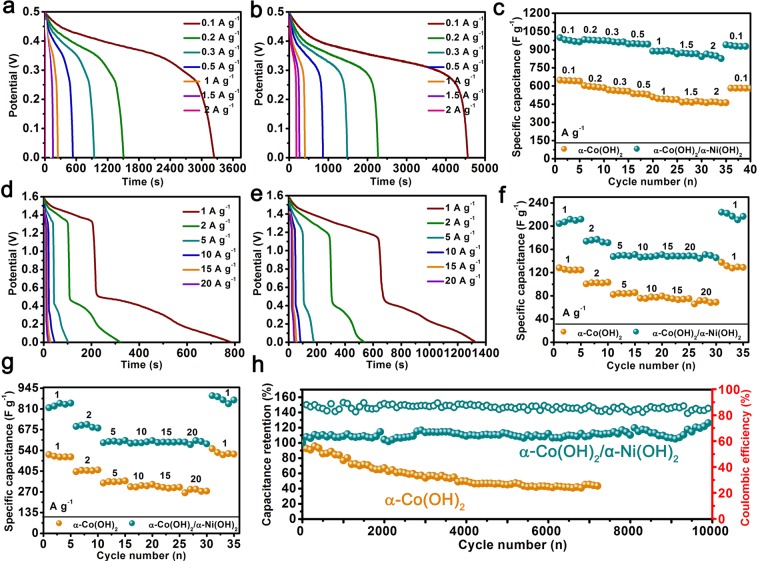
GDC, rate performance test and cycle test of α-Co(OH)_2_ nanowires and α-Co(OH)_2_/α-Ni(OH)_2_ nanorods, respectively. (**a**,**b**) Discharge curves in three-electrode system (**c**) their rate performance in three-electrode system, (**d**,**e**) the discharge curves in two-electrode system, (**f**), rate performance in two-electrode system, (**g**) rate performance of single electrode of two materials in two-electrode system, (**h**) cycling stability and coulombic efficiency of α-Co(OH)_2_ nanowires and α-Co(OH)_2_/α-Ni(OH)_2_ nanorods.

The electrochemical performances are further proved by Galvanostatic discharge-charge (GDC), rate performance test and cycle test. Figure (5a,b) display the discharge curves of α-Co(OH)_2_ nanowires and α-Co(OH)_2_/α-Ni(OH)_2_ heterojunction nanorods from a potential of 0–0.5 V at 0.1 to 2 A g^−1^ of current densities in three-electrode system. The curves of the α-Co(OH)_2_ and α-Co(OH)_2_/α-Ni(OH)_2_ heterojunction all show the characteristics of the battery-like discharge platform during the discharge process, which further indicates that the charge storage of two materials mainly comes from the reversible redox reaction on surface of the electrode material. Comparing the discharge time of α-Co(OH)_2_ with that of the α-Co(OH)_2_/α-Ni(OH)_2_ heterojunction further, it is found that advantage of heterojunction materials is that they can release higher charges, which could be due to the complementary advantages of high ions transport in two-dimensional layered crystal structure and fast electron transport in α-Co(OH)_2_. According to GDC curves, the equation which is applied to calculate the mass specific capacitances of electrode is as follows^47,48^:

4 $${\rm{C}}={\rm{I}}{\rm{\Delta }}{\rm{t}}/{\rm{m}}{\rm{\Delta }}{\rm{V}}$$

where C is the specific capacitance of single electrode, I (A) is the discharge current, Δt (s) is discharge time, m (g) is the mass of active material, and ΔV (V) is the potential window of measurement.

The specific capacitances at different current density are show in Fig. 5c. The specific capacitance of α-Co(OH)_2_ nanowires are calculated by formula (4). The value of specific capacitance is 648.3, 597.6, 567.1, 536.0, 496.7, 475.3 and 469.8 F g^−1^ at current density of 0.1, 0.2, 0.3, 0.5, 1, 1.5 and 2 A g^−1^, respectively, and which retains 72.5% of its initial specific capacitance. When the current density returns to 0.1 A g^−1^, the specific capacitance still holds high initial capacitance retention with 90%. For heterojunction α-Co(OH)_2_/α-Ni(OH)_2_ nanorods, which show a specific capacitance with 981.5, 979.2, 964.7, 950.3, 887.3, 872.85 and 854.3 F g^−1^ at same current density with α-Co(OH)_2_, respectively, yielding a 87% of initial capacitance-retention ratio, 95.2% capacitance retention is still kept when the material undergo rate cycle from high current density. The α-Co(OH)_2_/α-Ni(OH)_2_ heterojunction exhibit much higher specific capacitance, rate performance and stability than that of pure α-Co(OH)_2_.

In addition, in order to assess the performance of electrode material in practical applications, the α-Co(OH)_2_ and α-Co(OH)_2_/α-Ni(OH)_2_ heterojunction were used as positive materials and AC were chosen as negative materials to assembled to two-electrode device (ASC). The GDC curves of α-Co(OH)_2_ and α-Co(OH)_2_/α-Ni(OH)_2_ are shown in Fig. 5d,e, respectively, and two discharge voltage plateaus can be observed in the voltage window of 0–1.6 V, which indicate the discharge behavior mainly comes from pseudocapacitances. Moreover, discharge time of the heterojunction α-Co(OH)_2_/α-Ni(OH)_2_//AC device is significantly higher than single α-Co(OH)_2_. The capacitance of heterojunctionα-Co(OH)_2_/α-Ni(OH)_2_//AC and α-Co(OH)_2_//AC device can be computed to be 207.2, 175.8, 151.1, 150.9, 147.8, 145.6 (70.2% of capacity retention) and 124.4, 102.4, 84.2, 77.8, 74.1, 71.8 F g^−1^ (57.7% of capacity retention) at current density of 1, 2, 5, 10, 15 and 20 A g^−1^ by GCD cures, respectively. The heterojunction α-Co(OH)_2_/α-Ni(OH)_2_//AC devices display much higher specific capacitance and rate performance, which are attributed to the complementary advantages of large ions contact in intercalated structure and high electron rate of α-Co(OH)_2_ electrode, well contact between material and current collector and the unique surface morphology with plicated structure, leading to a high utilization. For a comparison, the Fig. 5g provide the rate capacitance of α-Co(OH)_2_/α-Ni(OH)_2_ heterojunction single electrode and α-Co(OH)_2_ single electrode in device. The values in heterojunction are 828.8, 703.2, 604.4, 603.6, 591.2, 582.4 F g^−1^ and 609.6, 527.2, 452.5, 422.8, 410.4, 404.8 F g^−1^ from 1 to 20 A g^−1^, respectively. The value (828.8) is close to capacitance of three-electrode system at 1 A g^−1^ current density, further indicating the heterojunction achieve a high utilization because of construction of the high-efficiency ion and speedy electron transmission.

In order to prove that the electrode exhibit excellent performance. Table 1 lists the comparison between this work and some Ni(OH)_2_-based or Co(OH)_2_-based in previous literature, which can be reflected that the values in three-electrode system are lower than those reported, but a highest specific capacitance is displayed in two-electrode system, implying a enough utilization of the electrode.

**Table 1:** Comparison of the capacitance between this work and some reported Ni(OH)_2_-based or Co(OH)_2_ based.

Electrode material	Capacitance retention rate of single electrode@Current density (From three-electrode system to two-electrode system)	Reference
Ni(OH)_2_-Co(OH)_2_	31.2%@1 A g^−1^	^11^
Co-Ni(OH)_2_/Ni_3_S_2_	18.0%@1 A g^−1^	^22^
NiCo_2_S_3_@Ni(OH)_2_@ppy	14.3%@1.56 A g^−1^	^26^
Ni(OH)_2_	18.4%@1 A g^−1^	^52^
Co_3_O_4_/CoS/Ni(OH)_2_@Co	26.19%@10 mA cm^−2^	^53^
Ni(OH)_2_/Mn_2_O_3_	20.6%@1 A g^−1^	^54^
Ni_3_S_2_@ Co(OH)_2_@Ni	20.5%@0.33 A g^−1^	^58^
Ni(OH)_2_	32.0%@1 A g^−1^	^59^
NiCoP @C@Ni(OH)_2_	22.4%@1 A g^−1^	^55^
(Ni-Co-S)/Co(OH)_2_	35.2%@1 A g^−1^	^56^
CoMoO_4_@Ni(OH)_2_	61.7%@1 A g^−1^	^60^
RGO/α-Ni(OH)_2_	22.5%@1 A g^−1^	^61^
Ni-Co-S/G	32.7%@1 A g^−1^	^57^
FeOF/Ni(OH)_2_	27.7%@1 A g^−1^	^62^
Mg(OH)_2_/Ni(OH)_2_	35.1%@1 A g^−1^	^63^
Co-α-Ni(OH)_2_/RGO	17.1%@1 A g^−1^	^64^
MWCNT/amor-Ni(OH)_2_/PEDOT:PSS	22.0%@5 mV s^−1^	^65^
α-Co(OH)_2_@α-Ni(OH)_2_	93.4%@1 A g^−1^	This work

**Figure 6:**
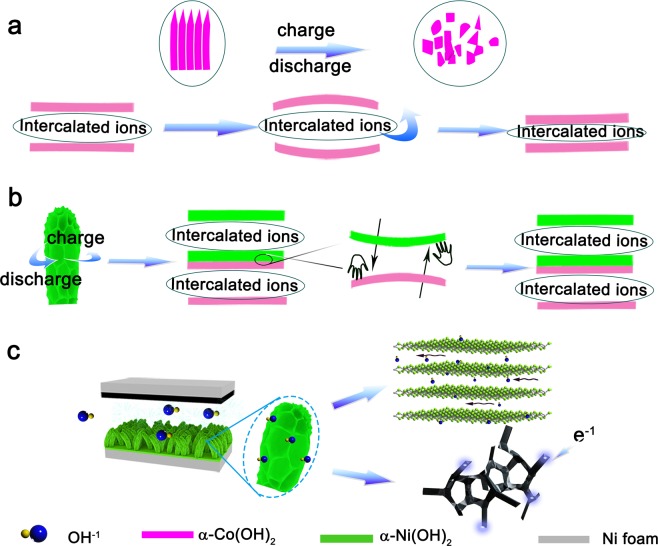
(**a**) Explanation of poor cycle life of α-Co(OH)_2_. (**b**) The illustration diagram of excellent stability of α-Co(OH)_2_/α-Ni(OH)_2_ heterojunction, (**c**) abridged general view of α-Co(OH)_2_/α-Ni(OH)_2_ heterojunction with high performance.

In the cycling test work, specific capacitance of α-Co(OH)_2_//AC device display decaying trend as the number of cycles increases at 5 A g^−1^ (Fig. 5h). This unsatisfactory phenomenon may be caused by the strain induced due to the anions breaking away from the interlayer, which leads to the collapse of the structure. In order to further verify hypothesis, morphology of the electrode material was tested after 7200 cycles of charge/discharge and the structure of the nanowires have been transformed to compacted bulk (Fig. S3). In order to better understand the change of two-dimensional layered structure of α-Co(OH)_2_ in charging and discharging process, The diagram is shown in Fig. 6a. Encouragingly, the α-Co(OH)_2_/α-Ni(OH)_2_ heterojunction complexes achieve an outstanding cycle stability after 10000 cycles at 5 A g^−1^ and a capacitance retention was up to 123.6%. An improvement of capacitance for hybrid material can be regard as activation of the active sites. This stable structure can be attributed to the mutual-support deformation stress between the layered structures of α-Ni(OH)_2_ and α-Co(OH)_2_ effectively organized a stable heterojunction (Fig. 6b). The morphology of the heterojunction after 10000 cycles is further analyzed by SEM (Fig. S4), demonstrating well initial structure of α-Co(OH)_2_/α-Ni(OH)_2_ heterojunction material, which confirm the above conclusion. In addition, the heterojunction also delivers a high coulombic efficiency of 85.3%. The illustration diagram in Fig. 6c shows excellent performance which could be attributed to the synergistic complementarity of the microstructure in two aspects. Firstly, it realizes high concentration ion transport within interlaminar structures and rapid electron transport of α-Co(OH)_2_. Besides, fast transport channels by α-Co(OH)_2_/α-Ni(OH)_2_ heterojunction and Ni foam of 3D conductive network are constructed with large numbers of ions contacting with electrode materials.

**Figure 7:**
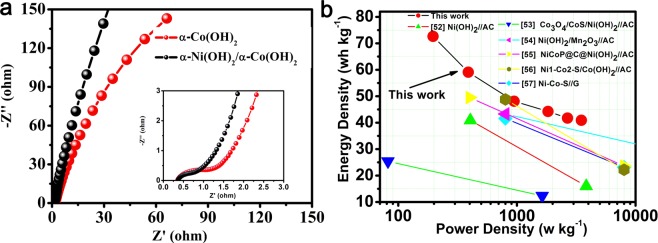
(**a**) EIS of the α-Co(OH)_2_/α-Ni(OH)_2_ and α-Co(OH)_2_. (**b**) Their Ragone plot.

The electrochemical impedance spectroscopy (EIS) of the α- Co(OH)_2_//AC and α-Co(OH)_2_/α-Ni(OH)_2_//AC device were analyzed (0.01 to 10^5^ Hz), as shown in Fig. 7a. The intersecting point with the real axis region can be regarded as conjunct resistance (Rs) in the high-frequency, including inherent resistance of the active materials, ionic resistance of electrolyte, and contact resistance between active material and current collector^49–51^. The Rs values of the α-Co(OH)_2_ and α-Co(OH)_2_/α-Ni(OH)_2_ heterojunction are about 0.35 Ω and 0.36 Ω, respectively, illustrating high conductivity and small contact impedance. The high-frequency semi-circle (Rct) is attributed to the charge-transfer process of Faradic reactions locating at the electrode-electrolyte. The half-ring radius of α-Co(OH)_2_/α-Ni(OH)_2_ heterojunction material at high frequencies is smaller than that of α-Co(OH)_2_ itself, which indicate the rapid electronic transfer within the material. The slope of straight in low-frequency presents Warburg resistance (Zw), which can be interpreted as the result of diffusion of OH^−1^ ion in the electrolyte. The Angle between the straight lines of low-frequency region of α-Co(OH)_2_/α-Ni(OH)_2_ heterojunction material and the X-axis is close to 90°, which testifies the higher ion transfer rate on the surface of the electrode material. The reason for the low transmission impedance is that the construction of α-Co(OH)_2_/α-Ni(OH)_2_ heterojunction enhances the complementary advantages of ion and electron transport in material each other, as well as the construction of continuous conductive skeleton between heterojunction material and collecting fluid, and the large ion contact between material and electrolyte. The energy density (E) and power density (P) as crucial parameters are used to judge its practical application. The calculation formulas are listed below:

5 $${\rm{E}}=0.5\ast {\rm{C}}\ast {({\rm{\Delta }}{\rm{V}})}^{2}/3.6$$

6 $${\rm{P}}=3600\ast {\rm{E}}/{\rm{\Delta }}{\rm{V}}$$

where C represents the specific capacitance (F g^−1^), ΔV is the potential window in two-electrode system (1.6 V), Δt is the discharging duration (s) of the device.

The energy density and power density can be calculated by the formula listed above (Eqs 5 and 6), which are delivered in Fig. 7b. The Ragone plot of heterojunction α-Co(OH)_2_/α-Ni(OH)_2_//AC device reflects a high energy density values of 72.6 Wh kg^−1^ at power density of 196.4 W kg^−1^ and energy density of 40.9 Wh kg^−1^ at power density of 3491.8 W kg^−1^, which are obviously higher than Ni(OH)_2_//AC (40.9 Wh kg^−1^ at 405 W kg^−1^)^52^, Co_3_O_4_/CoS/Ni(OH)_2_//AC (25.4 Wh kg^−1^ at 81.8 W kg^−1^)^53^, Ni(OH)_2_/Mn_2_O_3_//AC (41.6 Wh kg^−1^ at 799.4 W kg^−1^)^54^, NiCoP@C/Ni(OH)_2_//AC (49.5 Wh kg^−1^ at 399.8 W kg^−1^)^55^, Ni1-Co2-S/Co(OH)_2_//AC (48.8 Wh kg^−1^ at 800 W kg^−1^)^56^ and Ni-Co-S//G (43.3 Wh kg^−1^ at 800 W kg^−1^)^57^. All these results prove an enormous potential to apply the heterojunction material for low cost energy storage device.

## Conclusion

In summary, the α-Co(OH)_2_/α-Ni(OH)_2_ heterojunction nanorods arrays on Ni foam is successfully synthesized by a facile and mild nanotechnology in large-scaled industrialization. The α-Co(OH)_2_/α-Ni(OH)_2_ heterojunction structure with two-dimensional crystal intercalation structure expands the complementary advantages of ion transport and electron transport between the two materials. Meanwhile, the heterojunction material also integrates the fluid-collecting device with high electron transport and utilizes the own advantages of nanomaterials, so that the heterojunction material can achieve a high utilization rate with a capacitance retention rate of 93.4% at 1 A g^−1^ for single electrode (based two-electrode system and three-electrode system) and long cycle life (the capacitance retention rate is 123.6% at 5 A g^−1^ for 10000 cycles). In addition, a good rate performance (70.2% of capacitance retention from 1 to 20 A g^−1^), a high energy density (72.6 Wh kg^−1^ at 196.4 W kg^−1^ and 40.9 Wh kg^−1^ at 3491.8 W kg^−1^) and cycling stability can be came down to the following ways: (1) The complementary advantages between abundant ion supply of α-Ni(OH)_2_ and ideal conductance of α-Co(OH)_2_ further enhance ions and electron transport rate. (2) The materials reveal plicate morphology with a large surface and a large intercalated structure from each material, which deepen utilization of active sites. (3) Ni foam as conductive substrates combine the steadily heterojunction structure constructs a continuous conductive frame, which further guarantees the rapid electron transport. (4) The α-Ni(OH)_2_ is compactly fixed to the surface of α-Co(OH)_2_, the mutual deformation stress ensures that the layered structure of the two materials can be stable to each other. The α-Co(OH)_2_/α-Ni(OH)_2_ heterojunction nanorods arrays on Ni foam open up a fascinating prospect for designing supercapacitor with excellent performance using a simple and mild method in larger-scale preparation.

## Supplementary information

Surpporting Imformation
