# Supplementary material for: Heterojunction α-Co(OH)2/α-Ni(OH)2 nanorods arrays on Ni foam with high utilization rate and excellent structure stability for high-performance supercapacitor
Source: Sci Rep. 2019 Sep 4;9:12727. doi: 10.1038/s41598-019-49138-5 (PMC6726609; doi:10.1038/s41598-019-49138-5)
Supplement: Supplementary file 1 — Surpporting Imformation [file 41598_2019_49138_MOESM1_ESM.docx]

**Supporting Informafion**

**Heterostructure α-Co(OH)_2_/α-Ni(OH)_2_ nanorods arrays on Ni foam with high utilization rate and excellent structure stability for high-performance supercapacitor**

Shaojie Zhou,^†^ Wutao Wei, ^†^ Yingying Zhang, ^†^ Shizhong Cui,^†^ Weihua Chen^‡,*^ and Liwei Mi^†,*^

†Center for Advanced Materials Research, Zhongyuan University of Technology, Zhengzhou, Henan 450007, P. R. China.

‡College of Chemistry and Molecular Engineering, Zhengzhou University, Zhengzhou, Henan 450001, P. R. China.

Correspondence and requests for materials should be addressed to W. C. (email: chenweih@zzu.edu.cn) or L. M. (email: mlwzzu@163.com).


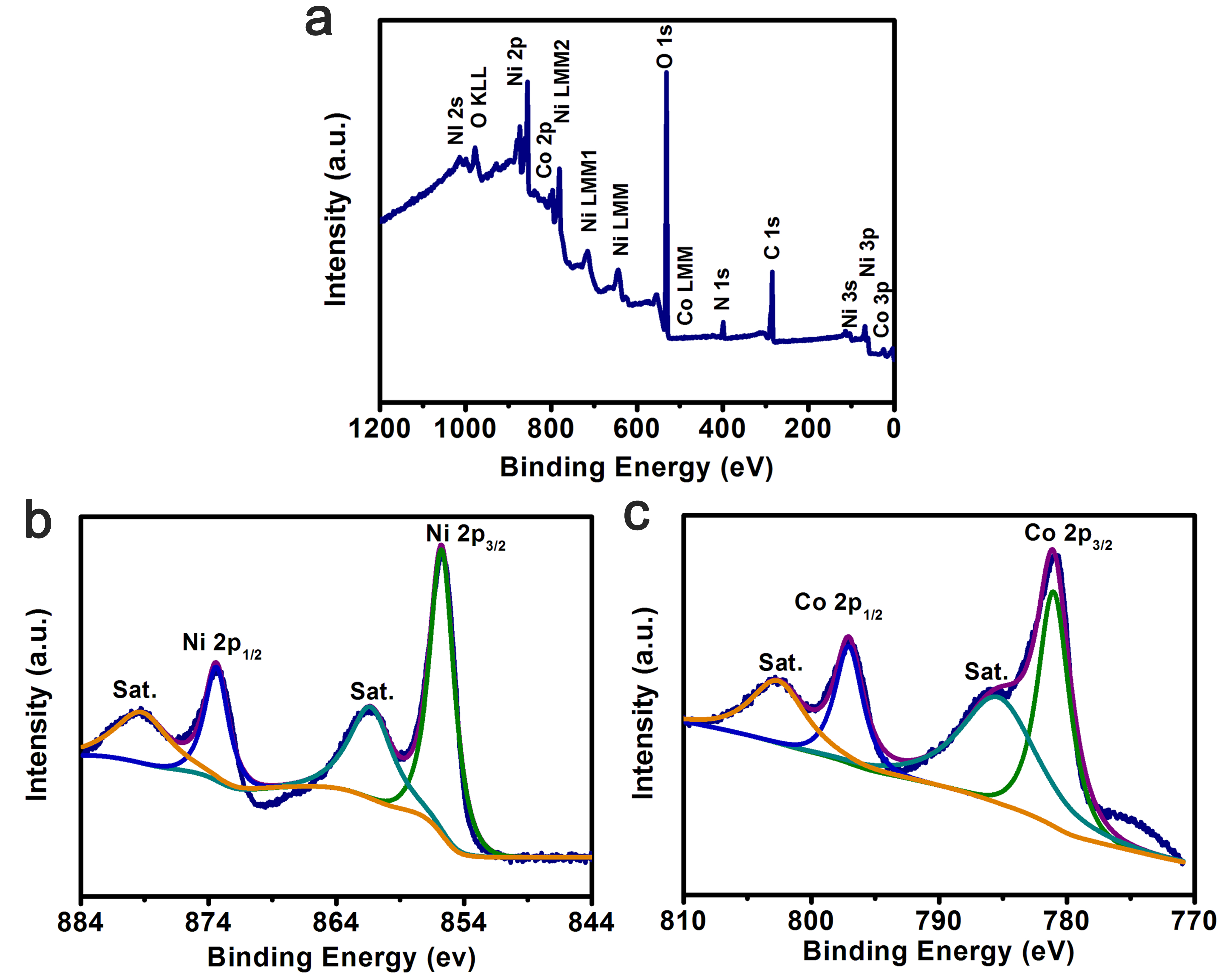


**Figure S1.** XPS spectra of heterojunction α-Co(OH)_2_/α-Ni(OH)_2_. (a) Broad scan, (b) Ni 2p XPS (c) Co 2p XPS.

The heterojunction was tested by XPS, as shown in Figure S1. The broad scan of heterojunction α-Co(OH)_2_ and α-Ni(OH)_2_ indicate existence of Ni, Co, C, N and O elements, which is consistent with EDS results (Figure S1a). Figure S1b show Ni 2p spectra, two strong peaks located at 855.8 and 873.4 eV, which are correspond to Ni 2p_3/2_ and Ni 2p_1/2_, respectively. In Co 2p XPS spectra (Figure S1c), the peaks at 855.9 and 873.3 eV are consistent with Co 2p_3/2_ and Co 2p_1/2_, respectively. These results indicate that the sample include α-Co(OH)_2_ and α-Ni(OH)_2_.


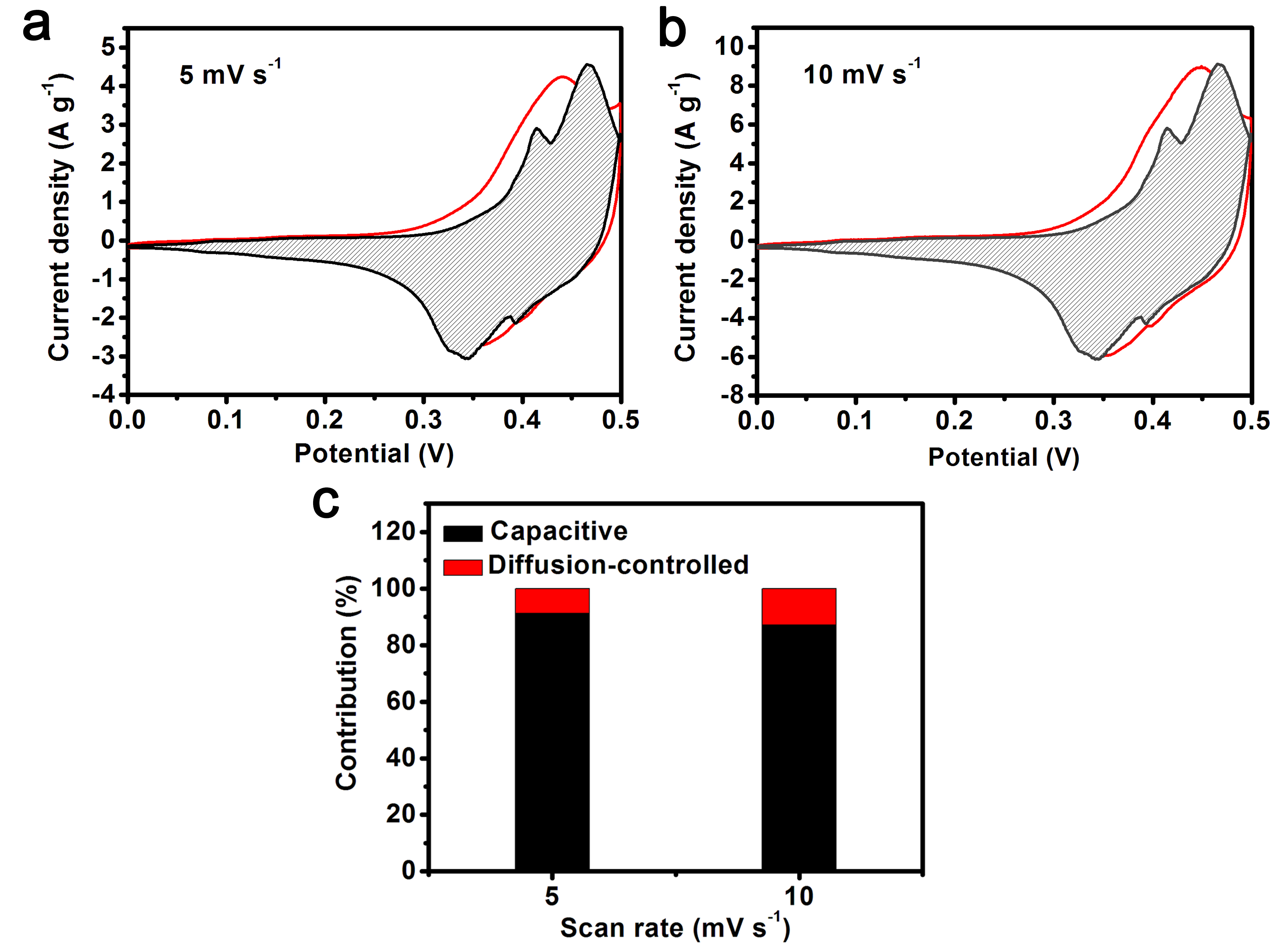


**Figure S2.** Analysis of capacitive and diffusion-controlled contributions in heterojunction α-Co(OH)_2_/α-Ni(OH)_2_. (a) 5 mv s^-1^, (b) 10 mv s^-1^, (c) corresponding contribution ratio at different scanning speed.

The total capacity can be divided into the capacitive (k1) and diffusion (k2)-controlled contributions. The total capacity can be calculated using the following formula:

i(V)=k_1_ν+k_2_ν^1/2^ (1)

To facilitate calculation, the formula can be converted to

i(V)/ ν^1/2^ =k_1_ ν^1/2^+k_2_ (2)

Where ν is the scanning speed, i is current. By plotting the relationship between ν^1/2^ and i(V)/ ν^1/2^, the k_1_ and k_2_ can be de determined. Figure S2a and Figure S2b shows the distribution of the capacitive and diffusion-controlled contributions at 5 mv s^-1^ and 10 mv s^-1^ in heterojunction α-Co(OH)_2_/α-Ni(OH)_2_ nanorod, respectively. As shown in Figure S2c，the capacitance contributes 91.2% and 87.1% of the total capacity at 5 and 10 mv s^-1^, respectively. The above results indicate that capacitive charge-storage dominates the mechanism in heterojunction materials.


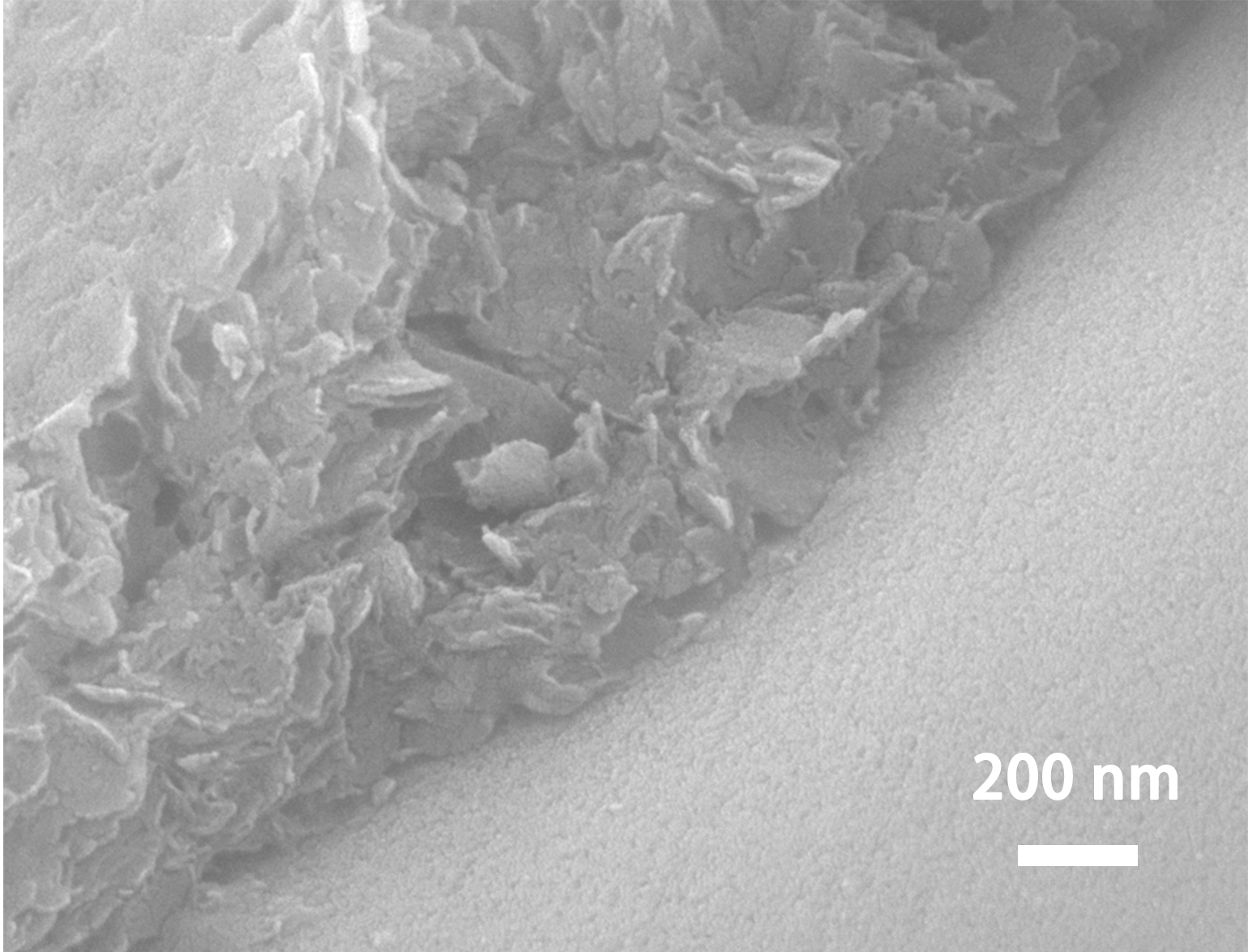


**Figure S3.** After 7200 cycles, the SEM of Co(OH)_2_.


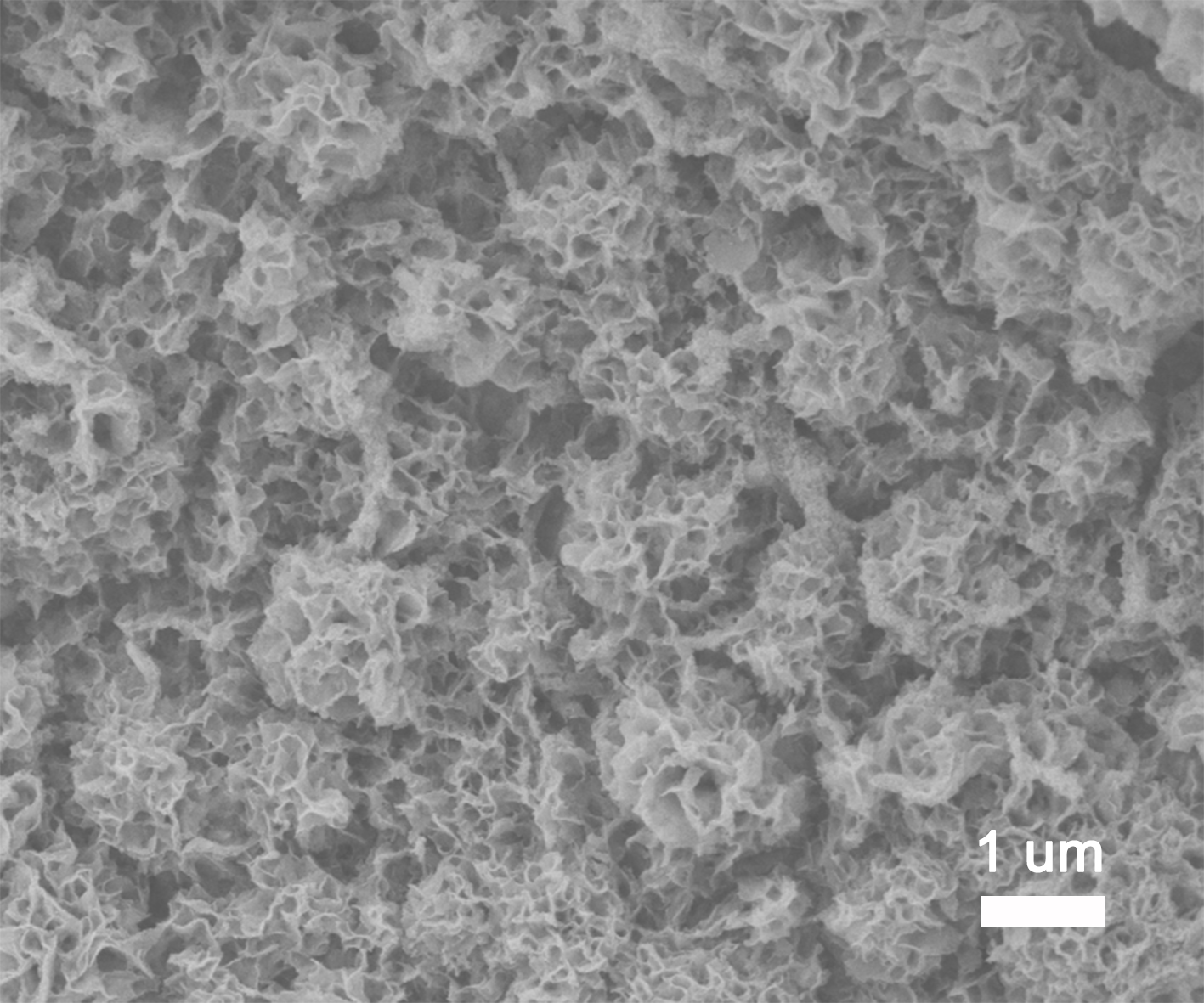


**Figure S4.** After 10000 cycles, the SEM of heterojunction α-Co(OH)@α-Ni(OH)_2_ nanorods arrays.
